# Preventing compulsory admission to psychiatric inpatient care through psycho-education and crisis focused monitoring

**DOI:** 10.1186/1471-244X-12-136

**Published:** 2012-09-05

**Authors:** Barbara Lay, Hans Joachim Salize, Harald Dressing, Nicolas Rüsch, Thekla Schönenberger, Monika Bühlmann, Marco Bleiker, Silke Lengler, Lena Korinth, Wulf Rössler

**Affiliations:** 1 Psychiatric University Hospital Zurich, Zurich, Switzerland; 2 Central Institute of Mental Health, Mannheim, Germany

**Keywords:** Involuntary placement, Psychiatric hospitalisation, Preventive monitoring, Crisis card, Randomised controlled trial

## Abstract

**Background:**

The high number of involuntary placements of people with mental disorders in Switzerland and other European countries constitutes a major public health issue. In view of the ethical and personal relevance of compulsory admission for the patients concerned and given the far-reaching effects in terms of health care costs, innovative interventions to improve the current situation are much needed. A number of promising approaches to prevent involuntary placements have been proposed that target continuity of care by increasing self-management skills of patients. However, the effectiveness of such interventions in terms of more robust criteria (e.g., admission rates) has not been sufficiently analysed in larger study samples. The current study aims to evaluate an intervention programme for patients at high risk of compulsory admission to psychiatric hospitals. Effectiveness will be assessed in terms of a reduced number of psychiatric hospitalisations and days of inpatient care in connection with involuntary psychiatric admissions as well as in terms of cost-containment in inpatient mental health care. The intervention furthermore intends to reduce the degree of patients’ perceived coercion and to increase patient satisfaction, their quality of life and empowerment.

**Methods/Design:**

This paper describes the design of a randomised controlled intervention study conducted currently at four psychiatric hospitals in the Canton of Zurich. The intervention programme consists of individualised psycho-education focusing on behaviours prior to and during illness-related crisis, the distribution of a crisis card and, after inpatient admission, a 24-month preventive monitoring of individual risk factors for compulsory re-admission to hospital. All measures are provided by a mental health care worker who maintains permanent contact to the patient over the course of the study. In order to prove its effectiveness the intervention programme will be compared with standard care procedures (control group). 200 patients each will be assigned to the intervention group or to the control group. Detailed follow-up assessments of service use, psychopathology and patient perceptions are scheduled 12 and 24 months after discharge.

**Discussion:**

Innovative interventions have to be established to prevent patients with mental disorders from undergoing the experience of compulsory admission and, with regard to society as a whole, to reduce the costs of health care (and detention). The current study will allow for a prospective analysis of the effectiveness of an intervention programme, providing insight into processes and factors that determine involuntary placement.

**Trial registration:**

Current Controlled Trials ISRCTN63162737.

## Background

Compulsory admission and use of coercive measures are accepted as necessary (and justified by the laws of most countries) in certain situations in clinical psychiatry: Compulsory admission is mandatory in cases of considerable danger to oneself or to others due to psychiatric illness. Notwithstanding that compulsory admission is regarded as indispensable to cope with violence and to prevent possible physical and psychological damage to the patient and/or others, the use and potential misuse of coercion in psychiatry has been accompanied for quite some time by critical debate [1]. A major argument in this debate is that coercive measures affect a patient’s personal interests profoundly: They constitute not only a serious restriction of a person’s freedom, but may also be perceived by a patient as unjustified or harmful [2]. Moreover, coercion may have adverse effects on the therapist-patient relationship and be associated with negative outcomes [3].

Beyond the ethical and personal relevance for the patients concerned, compulsory admission of people with mental disorder has far-reaching effects also in terms of health care costs. In most medical fields today, a primary aim of health care strategies is to prevent inpatient treatment and to minimise the length of hospital stays without diminishing the quality of care for the patient. This applies particularly to chronic diseases where institutionalised care affects the life of the patients to a maximum, while at the same time significantly increasing the financial burden of the funding agencies.

The legal basis for compulsory admission in Switzerland is regulated by the Swiss Civil Code Art. ZGB 397a, but implementary regulations are on a Federal State (“cantonal”) level. Unlike in other countries, there is no involuntary outpatient commitment in Switzerland. According to Art. 397a ZGB “an adult or a ward of court may be committed to or detained in a suitable institution on account of mental illness, learning disabilities, alcoholism, addiction to other substances or serious self-neglect, provided there is no other way to ensure his personal welfare. Account must be taken of any strain the person places on those around him or her. The person in question must be released as soon as his condition allows” [4]. Decisions on committal are taken by the guardianship authority at the domicile of the person concerned or, where there is risk in delay, by the guardianship authority of the place where he is staying. In cases where there is risk in delay or the person is psychologically ill, the cantons may authorise other suitable bodies to take such decisions. In the Canton of Zurich, all physicians are authorised to mandate compulsory admission to psychiatric care.

According to international comparisons, the frequency of involuntary placement has increased in a number of European countries, whereas in most EU member states involuntary placement quotas have remained more or less stable [5]. Compared to other European countries, Switzerland has one of the highest rates of compulsory admission of people with mental disorder [6]: Of all inpatient psychiatric admissions in the Canton of Zurich, compulsory admissions accounted for more than 30% for many years (Austria 19%; Germany 15%; Portugal 3% [7]). This rate has declined to some extent in recent years, but is at a comparatively high level until today (23% in 2007; PSYREC [8]).

In Switzerland though there is no shortage of community services: In the Canton of Zurich community mental health services are provided by 12 psychiatric institutions, and a total of 579 psychiatrists in office practice serve a population of 1.3 million people [9]. Despite extensive health care resources, the treatments offered are obviously not optimally adapted to the needs of mentally ill people.

In light of the high use of involuntary placement the need for innovative interventions to improve the current situation has frequently been advocated. Various efforts have been directed to integrate mentally disordered patients into the complex system of mental health services: These efforts range from empowerment and psycho-education to preventive measures targeting the “continuity of care”, which are considered to be helpful to enhance compliance with psychiatric treatment programmes, thus resulting in a reduction of voluntary and involuntary inpatient episodes, costs and transfers of patients to the criminal justice system.

A recent study reported promising results of a trial implementing a form of *advance agreement*. In a sample of 160 participants with a diagnosis of psychotic or bipolar disorder, the use of a joint crisis plan that was formulated by the patient, care coordinator, psychiatrist and project worker resulted in a significant decrease in the number of compulsory admissions. The number of days during which the patient was compulsorily treated was reduced by half [10]. The intervention also proved to be cost-effective [11]. Since the study sample was recruited from community mental health teams and included only a small diagnostic spectrum, results can not necessarily be generalised to psychiatry patients. Negative effects in terms of subsequent readmissions and self-efficacy at follow-up, however, have been reported by Papageorgiou et al., who studied the effects of advance directives in a sample of 156 in-patients receiving compulsory psychiatric treatment [12].

Furthermore, psycho-educational programmes have been introduced into routine psychiatric treatment and can be considered as a quality standard in psychiatric care [13]. Undoubtedly valuable and indispensable measures, their effect with regard to the above-mentioned specific problems, however, seems to be limited. From the perspective of *health psychology* a promising approach to prevent involuntary placement stresses the patient’s own interest to avert losing autonomy. This concept proposed by Krischke [14] suggests psycho-education focused on individual risk factors for crises and close monitoring by a personal mental health care worker in order to detect early signs of a crisis or threatening relapse, thus offering opportunities to react adequately and timely. A prerequisite of such an approach is the availability of crisis centres within the system of community mental health services which patients can contact in case of a threatening relapse.

Another concept originating in the voluntary sector as an advocacy tool for use in mental health emergencies is that of C*risis Cards*[15]. Crisis cards allow patients to state how they wish to be treated in a mental health emergency, during which they may have difficulties in making their wishes known. A crisis card may provide information about contact persons the patient knows and trusts, current treatments or treatments that have proved helpful or unhelpful in the past, or any medication the patient is currently taking.

Although the topic is of high relevance for the patients concerned as well as for health care policy, to date there is only preliminary research in this field. Effects in terms of more robust effectiveness criteria (e.g. significant reduction of admission rates, cost-effectiveness) have been investigated in larger study samples only lately:

A randomised controlled trial, CRIMSON, is currently conducted in Great Britain [16]. This trial aims to determine whether joint crisis plans, compared with treatment as usual, are effective in reducing the proportion of mental-health-service-users treated or detained under UK’s Mental Health act and will lead to improvement in total cost of care provision as well as further secondary outcomes. Eligible service users are people with a history of relapsing psychotic illness.

A further ongoing randomised controlled trial in the Netherlands aims to study the differential effect of two types of crisis plans (a crisis plan facilitated through the patient’s advocate, or through the clinician), compared to a control condition [17]. This study investigates effects on the number of psychiatric emergency visits and (involuntary) admissions and on social and psychological functioning in adult outpatients with psychotic or bipolar disorder. Moreover, it addresses the mediating mechanisms responsible for possible effects, such as the quality of the therapeutic alliance, therapy adherence, self-efficacy and insight into illness.

A randomised controlled trial with a similar design as the study described in this paper was just finished at Central Institute of Mental Health Mannheim, Germany (PRÄVENT-study). However, no results were published to date. Due to the similar approach, findings of the PRÄVENT-Study and this study may be directly compared in the future.

The objective of the present study is to implement and analyse the effectiveness of a 24-month intervention programme aiming to prevent compulsory admission of mentally ill patients to psychiatric inpatient treatment and to reduce the length of stays in connection with involuntary inpatient episodes. The specific interventions include measures which are likely to increase the self-management skills of chronically ill patients or which may increase their treatment adherence or pharmacological drug compliance.

The study is implemented as a subproject within the framework of the *Zurich Programme for Sustainable Development of Mental Health Services* (ZInEP)*.* ZInEP, which is funded by a private donation, is settled in the interface between research and supply. It consists of six different subprojects, each covering a specific psychiatric research field. The new projects of supply ought to become reliable points of reference for the further development of the psychiatric supply in Switzerland as well as in Europe, and contribute to a sustainable quality improvement.

## Methods

### Aims

The study has three main goals:

1) To evaluate the effectiveness of an intervention that aims to prevent compulsory admission to psychiatric hospital treatment and to reduce the time in hospital in connection with compulsory inpatient episodes (primary outcome criterion)

2) To evaluate changes in the perspective of the patients, hypothesizing that the intervention changes patients’ perceived coercion so that they feel less coerced and increases patients’ empowerment, quality of life and treatment satisfaction (secondary outcome criteria)

3) To examine which characteristics on the patient level (treatment history, diagnosis, social network, treatment adherence in the two-year follow-up) modify the outcome.

### Intervention

The main interventions of the study include:

(a) a programme of individualised psycho-educational instruction focusing on behaviours prior to and during a illness related crisis,

(b) the distribution of an individualised crisis card containing essential information and guidelines for preventing an acute crisis or for acting properly prior to or during a relapse, and

(c) a 24-month preventive monitoring of individual risk factors of relapse or inadequate disorder-treatment-related behaviour.

All measures are provided by a personal mental health care worker (psychologist), who will be available and maintain permanent contact to the patient throughout the intervention and follow-up period of the study.

The intervention programme shall not replace the patients’ regular therapy. Rather, it is considered as a supplementary measure to give chronically mentally ill patients individual support to become more actively involved in their care. Because the programme targets an increase in the patient’s empowerment, no structured collaboration between the personal mental health care worker and the regular treatment team is intended. The regular (inpatient) treatment team is informed that a patient is participating in the study. Members of the regular treatment team, however, are not aware of the outcome of randomisation.

(a) Individualised psycho-educational instruction of study patients

Immediately after recruitment and randomisation, each study patient in the intervention group attends the initial (1–3) instruction sessions. These sessions address information on relapses in mental disorders, treatment and adequate behaviour to prevent a crisis. The sessions are scheduled prior to discharge from the index episode (inpatient stay in the study centres) and individually adapted to the specific needs, experiences and history of illness of the respective patient. The series of initial sessions aims to establish a good relationship between the patient and the personal mental health care worker who assesses and guides the patient through the phase of preventive monitoring. The issues that are addressed include: information on the course of the disorder in question and risk factors for relapses, the structure of mental health care networks and ways of optimised use of services and treatments (including psychotropic drugs).

Additionally, individual risk factors for a potential crisis (e.g. familial, work or financial problems, accommodation etc.) and protective factors in case of a crisis (e.g. familial, social resources, behaviour etc.) are assessed. These risk and protective factors constitute an individual crisis guideline (check-list) to be discussed at the regular monitoring contacts during the follow-up period to predict signs of a crisis.

Psycho-educational instruction aims to increase the patients’ self-management skills, to enhance their overall treatment compliance and intake of psychopharmacologic drugs and to activate the patients’ potential for secondary prevention of relapses.

(b) Individualised crisis card

Based on the essential parts of the individual crisis guideline (a), a crisis card is drawn up in collaboration with the patient and handed out to the patient during the last instruction session. Crisis cards contain information on the patient, professional or personal contact persons, treatment, medication, and an individual action plan in case of a crisis or relapse.

Apart from collecting information and discussing the patient’s individual condition, this procedure deepens the relationship between patients and their personal mental health care workers and enhances the basis for trusting contacts during the follow-up. Moreover, it increases the patient’s feeling of being actively involved in his/her care. Accordingly, it is left to the patient to provide his/her regular therapist with a copy of the crisis card.

(c) Preventive monitoring of individual risk factors for compulsory re-admission to hospital

After discharge from the inpatient index episode, each patient is contacted every fourth week by telephone. These contacts will cover the complete follow-up period (24 months). Patients, however, are not visited at home. If the study participant does not answer the phone, the personal mental health care worker will follow-up until he/she gets through to the study patient. At each contact, the present status of study patients is assessed using the individual risk factor checklist. In order to assess service utilisation other scales are applied (instruments, see below). Monthly contacts are designed to provide a dense individual pattern of the course of the illness and the utilisation of health care services. Preventive monitoring enables detection of early signs of a crisis or a threatening relapse and offers opportunities to discuss issues or to intervene in case of problems.

It is only in emergency situations in which a patient may constitute a danger to himself or others, (e.g., if a study participant reports suicidal thoughts, but refuses to see any mental health care professional or other persons) that the personal mental health care worker will adopt measures to protect the study participant. Patients are informed that in such a situation the confidentiality agreement will be ignored. It is not intended, however, that the personal mental health care worker who maintains contact to the patient during follow-up communicates with a patient’s regular therapists or other mental health care professionals. Only if a patient explicitly desires this, may the personal mental health care worker impart information.

The individual data on the course of illness and care will be used to analyse a possible association with the major outcomes of the study. The recording of involuntary admissions to psychiatric hospitals, forensic facilities or prison terms during the follow-up period will allow analysis of the complex interdependencies between individual illness-related features and the pathways to institutionalised care in these sectors. Additionally, service utilisation data will be transformed into cost information and used to analyse the potential cost-effectiveness of the major study intervention (compared to treatment as usual in the control group).

## Study design

**Figure 1:**
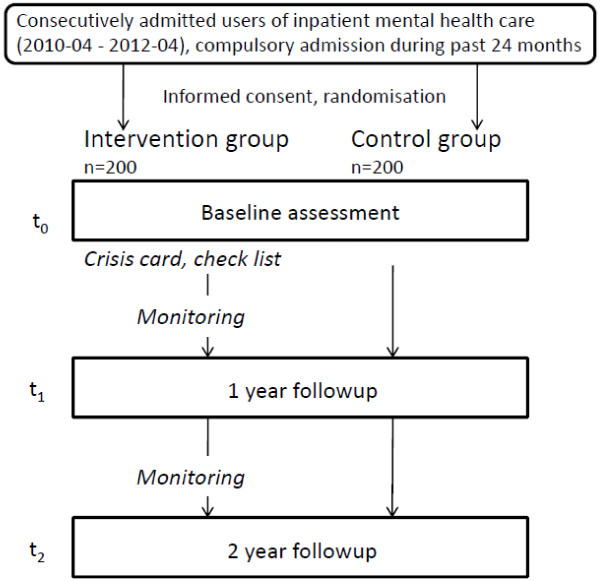
Flowchart.

The study is a prospective controlled trial. After provision of consent, the patients at each centre are assigned consecutively at random to either the intervention or the control group (Figure 1). To generate comparable groups and to prevent accidental bias as well as possible, block randomisation is used. A block size of 10 is chosen in order to ensure a balance in the numbers of subjects. Random allocation is made until the planned centre-specific sample sizes have been met.

The intervention is applied during a 24-month period. In both, the intervention and the control modality patients receive treatment as usual, i.e. regular out- or inpatient mental health care as offered in the respective study region.

The study encompasses a baseline assessment during the inpatient episode (t0) and a one-, respectively two-year follow-up (t1; t2). This applies to both the intervention and the control group. To enhance the compliance to contacts and assessments, each study participant (in the intervention and in the control group) receives an expense allowance of 60 CHF that is paid in instalments of 10 CHF at t0 and 25 CHF each at t1 and t2.

### Participants

The population from which the samples are recruited consists of consecutively admitted users of inpatient mental health care in the Canton of Zurich. Included are patients who

 – have been compulsorily admitted to psychiatry at least once during the past 24 months,

 – receive inpatient treatment in one of the four psychiatric hospitals participating in the study during the recruitment period (24 months),

 – are aged 18–65 years,

 – are residing in the Canton of Zurich,

 – and are willing and able to consent.

Patients who cannot be contacted by telephone (no telephone or mobile phone available) or who lack sufficient language skills are not eligible for inclusion.

Participation is not bound to a specific mental disorder. To be included in the study, however, patients must not have been diagnosed (index episode) with an organic mental disorder (ICD-10 F0), mental retardation (F7), a behavioural syndrome associated with physical factors (F5) or a developmental or behavioural disorder with onset usually occurring in childhood and adolescence (F8-F9).

The study sample is recruited from a naturalistic user sample from four study centres, all of them psychiatric hospitals mandated to provide psychiatric care to adult patients in the Canton of Zurich. Collaborating study centres are: Integrated Psychiatry Winterthur - Zürcher Unterland, Psychiatric Hospital Sanatorium Kilchberg AG, Clinic of Affective Disorders and General Psychiatry, Clinic for General and Social Psychiatry, Psychiatric University Hospital Zurich. At each study centre one designated mental health care worker is in charge of the study participants from that institution.

### Informed consent, data security and ethical approval

During the recruitment period all patients in the study centres fulfilling the inclusion criteria are informed (verbally and in written form) about the study and asked for their written consent prior to a planned discharge.

Personal data are treated according to the privacy regulations of the Psychiatric University Hospital Zurich, based on Art. 51 Personalgesetz (Human Resources Law). The results of the participant questionnaires and all interview data are accessible only to members of the study staff, but not to doctors or mental health care workers. The complete study protocol was endorsed by the Ethical Review Board for Clinical Studies of Canton Zurich, for Clinical Studies of Canton Zurich.

All study-related documents and data are stored on a protected central server of the ZInEP research group. Only members of the study staff have access to the respective files.

### Measurements

At baseline (t0) and again after 12 (follow-up t1) and 24 months respectively (follow-up t2), data on patient characteristics and health care use are gathered by means of face-to-face interviews and questionnaires. Interviews with study participants (intervention and control patients) are conducted by the members of the study staff.

Baseline assessment is scheduled upon discharge from the index episode at one of the study centres. Additional data concerning individual risk factors and service utilisation are collected during the monthly telephone contacts between the patient and his/her personal mental health care worker (intervention group).

As the overall number of questions at baseline assessment is high, a limited number of scales will be applied at the t1- and t2-follow-ups to limit the burden on patients and to comply with restricted time resources available for the interview session. Table 1 provides an overview of the measurements and the instruments used at the different time points.

**Table 1:** Outcome measures

**Variables**	**Instrument**	**Time of assessment**
Sociodemographic data	Patient file; CSSRI-EU	t0, t1, t2
Utilisation of mental health care or other relevant services (Previous health care use; number and length of voluntary and involuntary psychiatric inpatient episodes; frequency and length of stays in forensic facilities, frequency of use of outpatient psychiatric care and of other services during follow-up)	CSSRI-EU	t0, t1, t2 continuously in the intervention group; every 3 months in the control group
Psychopathology	CGI; GAF; OQ- 45; BPRS	t0, t1, t2
Risk of self-harm or threat to others	Patient file; HCR-20; PCL-SV; BPRS	t0, t1, t2
Informal coercion/perceived coercion	MacArthur AES	t0
Empowerment	Empowerment-Scale	t0, t1, t2
Treatment satisfaction	CSQ-8	t0, t1, t2
Quality of life	WHO-QoL-Bref	t0
Social support	BSSS	t0
Internalised Stigma	Internalised Stigma of Mental Illness Inventory	t0, t1, t2
Self-esteem; Cognitive appraisal of stigma as a stressor; Emotional reactions to compulsory admission	Rosenberg Self-Esteem Scale; Stigma-Stress Scale; 4 items	t0

#### Instruments

The instruments and scales applied in this study are widely used in international mental health care or clinical psychiatric research. All instruments are administered in their German version. The following dimensions are assessed:

##### Patient characteristics

Information regarding socio-demographic status, occupational, financial and living situation as well as clinical diagnoses is extracted from the patient file (index psychiatric episode) and supplemented by a comprehensive interview based on the Client Sociodemographic and Service Receipt Inventory CSSRI-EU [18].

##### Health care use

The CSSRI-EU is also used for a detailed assessment of the patient’s treatment history and treatment during follow-up. By means of this inventory, the frequency and lengths of (voluntary and involuntary) psychiatric inpatient episodes, the frequency and lengths of stay in forensic facilities, the frequency of use of outpatient psychiatric care, the amount and frequency of contacts to other health care professionals and the kinds and doses of psychopharmacological drugs taken are determined.

##### Psychopathology

is assessed using the Brief Psychiatric Rating Scale BPRS, extended version [19,20], which consists of 24 items, each rated on a 7-point scale based on a semi-structured interview guide.

Illness severity and global improvement or change are measured by the Clinical Global Impressions CGI [21]. To assess the patient’s global level of psychological, social and occupational functioning the Global Assessment of Functioning GAF [22] is applied as part of the clinical assessment.

Apart from clinical judgements the Outcome Questionnaire OQ-45.2 [23] is used for the patient’s self-assessment. This questionnaire comprising 45 items to be rated on a 5-point scale is designed to track the patient’s treatment response. Four domains of functioning are evaluated by the patient: symptoms of psychological disturbance, interpersonal problems, social role functioning and quality of life.

##### Risk of self-harm or threat to others

For a first screening of violent or self-harming behaviours in the past, information within the patient’s file prior to baseline consultation is checked. If the patient’s history is suggestive of such behaviours or the baseline interview brings out such behaviours, risk of self-harm or threat to others is rated by the staff members and if so, is assessed throughout the follow-up. Ratings are based on the Brief Psychiatric Rating Scale BPRS and a semi-structured interview developed by the research group Mental Health Services, CI Mannheim, based on the Violence Risk Assessment Scheme HCR-20 [24] and the screening version of the Hare Psychopathy Checklist PCL-SV [25]. The interview includes risk markers (32 items) which reflect past and current correlates of violence as well as situational factors that might aggravate or mitigate risk and are deemed to be predictors of recidivism, violence and the inability to respond to therapeutic intervention.

##### Perceived coercion / informal coercion

To assess the patients’ perceptions of coercion during their admission to psychiatric hospital, the MacArthur Admission Experience Survey AES [26] is applied (short form; 15 items). Additionally, perceptions of the fairness and effectiveness of coercive measures and methods of informal coercion are assessed [27,28]. Informal coercion includes negative pressures and threats as to the use of legal actions, as well as threats concerning the execution of payments, housing restrictions and the right to child custody. All coercion questions are presented on a dichotomous true/false scale. At t1 and t2 follow ups the MacArthur Admission Experience Scale, modified for outpatient use, will be applied.

##### Empowerment

The Empowerment-Scale [29] is used to measure self-efficacy, optimism, self-esteem and community-orientation. It consists of 28 statements on a 4-point scale that subjects can agree or disagree with.

##### Treatment satisfaction

is measured with the Client Satisfaction Questionnaire (CSQ-8; [30]). The patient’s global treatment satisfaction is assessed by the short form comprising 8 items.

##### Quality of life

is assessed by the Quality of Life Assessment Scale developed by the WHO, WHO-QoL-Bref, comprising 26 items (WHOQOL Group 1998; [31]).

##### Social support

To assess the possible impact of social ties and support on the outcome, the Berliner Social Support Scales BSSS [32] are used. The domains of support to be rated by the patient are: perceived available support (8 items), need for support (4 items), support seeking (5 items) and support actually received from the person closest to the patient (15 items).

##### Internalised stigma

Internalised stigma, or self-stigma, is assessed using the 29-item Internalised Stigma of Mental Illness Inventory [33]. Self-esteem, typically impaired by internalised stigma, is examined by the 10-item Rosenberg Self-Esteem Scale [34]. The cognitive appraisal of stigma as a stressor (i.e., whether perceived harm due to stigma exceeds perceived coping resources) is measured by the 8-item Stigma Stress scale [35,36]. Finally, four items assess shame and contempt as emotional reactions to compulsory admission.

#### Data preparation and data entry

Data entry is done by a research assistant at the coordinating centre. Quantitative data from all centres are entered immediately after data collection and checks of completeness and validity of data are performed regularly.

A cost catalogue for each service used by the patients will be compiled. To estimate comprehensive client-level care costs for each study patient, detailed information about frequency and duration of contact with health, social and other services provided by public organisations is recorded. Unit costs (per appointment, hospital per diem rate etc.) will be adjusted according to the frequency with and the duration for which each patient used each service as recorded in order to calculate the total cost of services and subtotals associated with various groups of component services and to compare the cost of support in standard care with that of the new intervention.

#### Data analysis and statistical power

All major endpoints of the study will be compared between the intervention and the control group. Primary outcome of the study is the time in hospital accumulated over all involuntary inpatient stays during the 24 month period. This outcome criterion will be analysed in terms of both “time in hospital” and savings of health care budgets (“health care costs”).

As further endpoints of the study (secondary outcome criteria) changes in the perspective of the patients are assessed. Hypothesised benefits of the intervention are a reduction of institutionalised care and coercion. Lower levels of subjectively experienced coercion are supposed to be associated with a respective increase in quality of life, treatment satisfaction and level of empowerment.

To address the major research questions, the quantitative outcome data will be analysed for the whole trial across the four centres, using General Linear Models. T-tests for independent samples are applied primarily to test the comparability of the intervention and the control group. Analyses of covariance are performed to compare the intervention and the control group with respect to the primary outcome (time in hospital accumulated over all involuntary inpatient stays) and secondary outcomes (reduction in levels of subjectively experienced coercion; increase in quality of life, treatment satisfaction and level of empowerment). Age, gender, centre and other variables which show significant between-group differences are included in the GLM models as covariates. To examine which characteristics on the patient level (treatment history, diagnosis, social network, treatment adherence in the two-year follow-up) modify the outcome, we examine the effects of these variables on the outcomes of interest within the intervention group, applying regression analyses. The level of significance is set at 0.05, two-tailed.

The planned overall sample size across all centres is 400 patients, of which 200 each will be assigned to the intervention group or to the control group. So each centre is expected to contribute 100 patients (50 intervention group and 50 control group).

Power analyses revealed that these sample sizes will be large enough to identify significant differences in the primary outcome criterion (length of compulsory inpatient episodes) between the intervention and the control group. For power analysis, length-of-stay data from the Psychiatric University Hospital Zurich over the period 2007–2008 were used. Compulsorily admitted patients (with the same diagnostic spectrum and age range as the sample considered for inclusion) stayed a mean length of 33 days (SD 42 days; CV 1.25) during a 24-month period. A log-normal distribution for the length of stay was assumed. A two-sample t-test on the mean ratio was performed to calculate the sample size on the basis of these data. The probability of type I error was set at 5% and probability of type II error at 20%. An 8–day reduction of the mean length of compulsory inpatient time due to the study intervention (approximating a quarter of inpatient time) was considered a clinically relevant difference. For confirming a reduction of the mean length of compulsory inpatient time from 33 days to 25 days a sample size of 193 in each (intervention and control) group would need to be treated (total sample size of 386).

## Discussion

The current study aims to prevent compulsory admission to psychiatric hospital and to reduce patients’ perceived coercion by offering an intervention programme consisting of individualised psycho-education and a 24-month preventive monitoring after inpatient admission. The intervention hereby intends to increase patient satisfaction, quality of life and empowerment in patients with serious mental disorders and previous compulsory psychiatric admission(s). Detailed follow-up assessments of service use, psychopathology and patients’ perceptions are scheduled 12 and 24 months after discharge. The intervention programme will be compared with standard care procedures in order to prove its effectiveness (in terms of “time in hospital” in connection with involuntary inpatient episodes, perceived coercion, empowerment, treatment satisfaction, quality of life) and cost-effectiveness (in terms of cost-containment in inpatient mental health care). Moreover the study seeks to identify individual and social factors that influence primary and secondary outcomes.

### Preliminary reflections on the limitations and strength of the study

A limitation of the study may be that long-term engagement in an intensive programme such as the one proposed may be difficult for patients with serious mental disorders. A selection bias at inclusion may be expected as a result of the consent procedure and during the follow-up as a result of dropouts. An advantage of the study, however, is that patients who have previously experienced compulsory measures in psychiatry may now have a strong interest to participate in such a programme in order to avoid further compulsory admission.

To determine dropout effects, differences between patients included in the study and those not included, but eligible for inclusion (to be traced by means of the respective hospital statistics) will be analysed. Likewise, patients who finish the programme and those who drop out during follow-up will be compared with respect to psychopathological, social and health care characteristics. We thus hope to better understand which patients might qualify for such a programme and to what extent results can be generalised. If dropout analysis does not reveal any systematic bias, the results likely may be generalised for a population of mentally ill at risk for involuntary psychiatric care.

Long-term engagement (24-month preventive monitoring), on the other hand, might be a strength of this study. Advance directives alone do not necessarily impact the outcome of care, as the study by Papageorgiou et al. [12] suggests. There are several reasons attributable to peculiarities on the part of the mental health care system, as well as on the part of patient behaviours, as to why directives might be ignored. It is considered a meaningful and integral part of this trial, therefore, to follow patients after hospital discharge, all the more so as a considerable number of this patient group holds reservations towards visiting an outpatient mental health professional. In this situation, a preventive monitoring may have beneficial effects, bringing to mind betimes the patient’s individual action plan in case of a crisis and motivating them to continue treatment.

A further general limitation relates to the fact that with such a programme only those factors that contribute to compulsory admission which are subject to the patient’s self management can be modified. Compulsory admission to a psychiatric hospital of course does not depend only on a patient’s clinical status and characteristics of his/her psychosocial background. Rather, service system aspects that determine referral or crisis intervention procedures might have a share in the use of involuntary placement. With the current intervention programme only risk factors that can be governed by the patients themselves might be reduced, whereas further steps would have to be taken in order to effect a change in service system aspects. The effectiveness of preventive measures focusing on compulsory admission so far has only rarely been studied in larger samples. A strong point of this study lies in the opportunity to conduct a prospective analysis using a controlled randomised design. In Switzerland, the number of compulsory admissions has remained at an exceedingly high level for a long time. This is an unacceptable situation which necessitates the testing and the analysis of interventions targeting the factors that might be amenable to change in order to reduce the rate of involuntary placements. The present study focuses on the continuity of care after discharge from psychiatric hospital, in particular on measures which may increase treatment adherence or pharmacological drug compliance. These measures, aiming at the activation of the patients’ potential for secondary prevention of relapses by increasing the self-management skills of chronically mentally ill patients, appear to be a new and innovative strategy to prevent involuntary inpatient episodes.

## Competing interests

The authors declare that they have no competing interests.

## Authors’ contributions

HJS, HD and WR conceived and designed the study with contributions from NR and BL. BL transformed the study protocol into this manuscript, implemented the project at the respective sites and manages the project. TS, MB, MB and SL have done the baseline assessments so far and are providing the intervention. LK is responsible for the data maintenance. HJS participates in the Steering Committee of the study and revised the manuscript critically. WR is head of the Research Department and leads all ZInEP projects. All authors read and approved the final manuscript.

## Pre-publication history

The pre-publication history for this paper can be accessed here:

http://www.biomedcentral.com/1471-244X/12/136/prepub
